# UVB-mediated down-regulation of proteasome in cultured human primary pterygium fibroblasts

**DOI:** 10.1186/s12886-018-0987-8

**Published:** 2018-12-18

**Authors:** Alexios J. Aletras, Ioannis Trilivas, Maria-Elpida Christopoulou, Sotiria Drakouli, Constantine D. Georgakopoulos, Nikolaos Pharmakakis

**Affiliations:** 1 Laboratory of Biochemistry, Department of Chemistry, University of Patras, 26 504 Patras, Greece; 2 Department of Opthalmology, Medical School, University of Patras, Patras, Greece; 3 Present address: Laboratory of Biochemistry, Faculty of Medicine, University of Thessaly, Volos, Greece

**Keywords:** Pterygium, Fibroblasts, Proteasome, UVB irradiation, Nrf2-ARE pathway, Src kinases

## Abstract

**Background:**

Pterygium is a condition characterized by epithelial overgrowth of the cornea, inflammatory cell infiltration and an abnormal extracellular matrix accumulation. Chronic UV exposure is considered as a pathogenic factor of this disease. Proteasome is an intracellular multi-subunit protease complex that degrades intracellular proteins. Among proteasome subunits the β5 (PSMB5), bearing chymotrypsin-like activity. It is considered as the main proteasome subunit and its expression is mediated by Nrf2-ARE pathway in many cell types. This study investigates the expression of PSMB5 in pterygium and the effect of UVB irradiation on its expression and activity in pterygium fibroblasts.

**Methods:**

Normal conjunctival and pterygium specimens were obtained from the bulbar conjunctiva of patients undergoing cataract surgery and from patients with pterygium undergoing surgical removal of primary tissue, respectively. Fibroblasts were isolated upon treatment of specimens with clostridium collagenase. The expression of PSMB5 and Nrf2 in tissues and cells was ascertained by RT-PCR analysis and western blotting. Cell survival was measured by the MTT method and the proteasome chymotrypsin-like activity was determined by fluorometry.

**Results:**

RT-PCR analysis showed that the expression of PSMB5 was significantly lower in pterygium than in normal conjunctiva. The expression of PSMB5 was mediated by the Nrf2/ARE pathway as indicated by using the Nrf2 activator Oltipraz. The expression of PSMB5 and Nrf2 by pterygium fibroblasts was suppressed in a dose dependent manner following UVB radiation of 0–50 mJ/cm^2^ doses. The expression of PSMB5, but not of Nrf2, remained at almost the control levels, when UVB exposure was performed after pre-incubation of cells with the src kinases inhibitor PP2. UVB irradiation had very low deleterious effect on fibroblasts survival, while it did not affect the proteasome chymotrypsin-like activity.

**Conclusion:**

In pterygium fibroblasts, UVB exposure leads to down-regulation of Nrf2/ARE-mediated PSMB5 gene expression, in which src kinases may be implicated. This effect may be partially responsible for the lower expression of PSMB5 detected in pterygium as compared to normal conjunctiva.

## Background

Pterygium is an eye disorder featuring hyperplasia of the cornea epithelium, usually bilateral, overgrowth of stromal fibroblasts and blood vessels, significant neovascularization, inflammatory cell infiltration and abnormal extracellular matrix accumulation, with elastin and collagen being the main components. Histopathological studies revealed that the pterygium epithelium, which invades the cornea, exhibits alterations such as squamous metaplasia and goblet cell overgrowth. An underlying damage of Bowman’s layer was also observed [1].

Immuohistologic studies indicate that pterygium is rather a limbal epithelial stem cell disorder, the formation and development of which may be triggered from a damage or activation of these stem cells [2]. The exact reasons of pterygium pathogenesis remain unclear, however ultraviolet (UV) radiation is considered to be the most acceptable cause for the initiation of this disorder [3, 4].

UVB radiation induces damage to cellular DNA, RNA, and extracellular matrix components through activation of different pathways [5]. UVB radiation also stimulates the expression of cytokines and growth factors in pterygium cells, which are considered to play pivotal role in the establishment and development of pterygium. The expression of cytokines and growth factors, such as IL-6, IL-8, TNF-α, bFGF, TGF-β and PDGF, in pterygium has been previously reported [6–10]. Extracellular matrix metalloproteinases (MMPs) may be also involved in the pathogenesis of pterygium. The expression of MMPs, such as MMP-2, MMP-3, MMP-9 and particularly MMP-1, which is the most abundant, has been demonstrated in pterygium tissues [11–13]. In addition, high expression of hypoxia inducible factor-1α (HIF-1α) and heat shock proteins (Hsp), such as Hsp90, Hsp70 and Hsp27 in pterygium tissues has been previously reported [14, 15]. They may contribute in the cellular defense mechanisms against stressful conditions .

The 26S proteasome is an intracellular proteolytic complex of two components: a 20S proteolytic core, which contains the catalytic activity, and a 19S regulatory subunit [16, 17]. The 20S proteolytic core is constructed from 28 subunits distributed in four heptameric rings stacked on top of each other, to form a structure resembling a barrel. The components of the two outside rings are called the α-subunits, and those of the two inside rings β-subunits. The proteasome possesses multiple protease activities, such as caspase-like, trypsin-like and chymotrypsin-like activity, localized on subunits β1, β2 and β5, respectively, and is able to degrade almost all the intracellular abnormal and denaturated proteins, as well as functional proteins, which have to be recycled. It also plays a role in cell cycle progression, and in cell development and death [18–21]. The normal function of proteasome and the consequent selective degradation of oxidized proteins that it exerts, highly contributes in the cellular defenses against oxidative stress. In contrast, the impaired function of the proteasome, leads to accumulation of oxidized/misfolded proteins within the cell, and may be responsible for the manifestation several degenerative diseases [22–26].

Previous studies have established that the expression of the catalytic subunits of the proteasome can be induced by various exogenous stimuli through the Nrf2-ARE (NF-E2 related factor 2-antioxidant response element) pathway and it has been proposed that the up-regulation of the proteasome subunits by Nrf2 activators may contribute to the protection of cells against oxidative damage through the selective degradation of oxidized proteins, thus preventing the formation of protein aggregates and their accumulation within cells [27–33].

Nrf2 belongs to family of the basic leucine zipper NF-E2 (nuclear factor erythroid-derived 2). Under normal conditions, it occurs as a complex with the protein KEAP1 (Kelch-like erythroid-cell-derived protein with CNC homology (ECH)-associated protein), remaining in the cytoplasm, where it is subsequently degraded by the ubiquitin/ proteasome pathway. Upon exposure to oxidative stress, the Nrf2-KEAP1 complex dissociates and Nrf2 migrates to the nucleus where interacts with small Maf-family proteins (small musculoaponeurotic fibrosarcoma TFs required for Nrf2 transactivation) forming heterodimers that bind to AREs in target gene promoters. Various small electrophile molecules, including oltipraz (5-[2-pyrazinyl]-4-methyl-1,2-dithiol-3-thione), inactivate KEAP1 and release the Nrf2, which then migrates to the nucleus [34].

The implication of proteasome in pterygium pathogenesis as well as the effect of UVB irradiation on its expression and activity in pterygium cells has not yet been studied. In the present study we investigated the effect of UVB irradiation on β5 proteasome subunit (PSMB5) expression and activity in pterygium fibroblasts.

## Methods

### Specimen selection

Specimens of normal bulbar conjunctival tissues were obtained from five patients (2 male and 3 female) undergoing cataract surgery, who did not show symptoms of an ocular surface disorder or of dry eyes, whose ages ranged from 61 to 90 years old (mean age ± SD 75.40 ± 12.26). Pterygium tissues located nasally were obtained from five patients (3 male and 2 female) subjected to primary pterygium excision surgery, whose ages ranged from 60 to 84 years old (mean age ± SD 71.80 ± 8.73). The study population was of Greek origin and all the specimens were collected in the Department of Ophthalmology, University Hospital of Patras, Patras, Greece.

### Conjunctival and primary Pterygium fibroblast cultures

Pterygium or normal conjunctival specimens immediately after excision were transferred on ice in the Laboratory (time of transfer ~ 10 min), where they were rinsed, minced and digested with 1 mg/ml clostridium collagenase in serum free Dulbecco’s modified Eagle’s medium (DMEM) (BioChrom AG, Berlin, Germany) for 2 h at 37 °C [35]. The released cells were pelleted, and re-suspended in DMEM supplemented with 10% heat inactivated fetal bovine serum (FBS), 1% penicillin and 1% streptomycin, plated and grown at 37 °C in a humidified 5% CO_2_ atmosphere. After overnight culture, non-adherent cells were removed and adherent cells were cultured in DMEM plus 10% FBS. For all experiments, cells were used after passage 4, at which time they comprised a homogeneous population of fibroblast like cells.

### UVB irradiation of cultured cells

Fibroblasts were seeded onto six well plates (Greiner, Frickenhausen, Germany) and grown in DMEM plus 10% FBS. Once the cells reached semi-confluence, the medium was aspirated and cells were starved in serum-free DMEM containing 0.2% lactalbumin hydrolysate (DMEM-0.2% LH) [36] for 24 h before the experiment. The medium was then replaced with PBS (1 mL/well) and monolayers were irradiated with 0 to 50 mJ/cm^2^ UVB light, using a Sankyo Denki G20T10E bulb, which emits UVB rays, with an emission spectrum ranging from 280 nm to 360 nm and one spectral peak at 306 nm. The exposure time (t) (in sec) was determined by the equation t = dose (mJ/cm^2^)/fluence rate (mW/cm^2^) [37]. After each exposure, cells were placed in fresh serum-free DMEM-0.2% LH and cultured for additional 24 h. When the Oltipraz (Sigma-Aldrich Chemical Co., St Louis, MO) or PP2 (Sigma-Aldrich Chemical Co., St Louis, MO) were used, the cells were pre-incubated in the presence of respective factor in serum-free DMEM-0.2% LH, for 2 h before the irradiation.

### Cell viability

Cell viability was detected by the MTT method, as previously described [38]. Briefly, after UVB radiation the cells were cultured for 24 h in DMEM-0.2% LH, then the conditioned medium was removed and a solution of 3-(4,5-dimethylthiazol-2-yl)-2,5-diphenyltetrazolium bromide (MTT) (Sigma-Aldrich Chemical Co., St Louis, MO) (1 mg/ml) in the same medium, was added. After incubation at 37 °C for 4 h the solution was thoroughly aspirated, the crystals of formazan were dissolved in DMSO and the absorbance at 540 nm was measured.

### Determination of proteasome activity

After UVB radiation the cells were cultured for 24 h in DMEM-0.2% LH, and then lysed with a solution of 1 mM DTT in water for 1 h at 4 °C [39]. Lysates were centrifuged at 14,000 g for 30 min at 4 °C and protein concentrations were determined in supernatants by the Bradford method [40], using bovine serum albumin as standard. Then the supernatants were assayed for the chymotrypsin-like activity of 20S proteasome with the hydrolysis of the fluorogenic substrate N-Succinyl-Leu-Leu-Val-Tyr-AMC (Sigma-Aldrich, Dorset, UK), for 1 h at 37^ο^C as previously described [41]. Proteasome activity was determined as the difference between the total activity of cell lysate supernatants and the remaining activity in the presence of proteasome inhibitor MG-132 at final concentration 100 μM. Fluorescence was measured, using a TECAN infinite M200 (Austria) fluorometer (excitation at 370 nm and emission at 440 nm).

### RNA isolation and RT-PCR analysis

Total RNA was extracted from normal conjunctival and pterygium tissue, as well as from fibroblasts as per the RNeasy spin mini-column manufacturer’s instructions (Qiagen, USA). RT-PCR analysis was performed at one step, using the One-Step RT-PCR kit (Qiagen, USA), according to the manufacturer’s instructions. RT was carried out for 30 min at 50 °C followed by a 15 min step at 95 °C. Amplifications were performed with 30 cycles. Each cycle included denaturation at 95 °C for 1 min, annealing at appropriate temperature for 1 min, extension at 72 °C for 1 min, followed by a final extension at 72 °C for 10 min. The PCR primers used for PSMB5, [(sense) 5′-GAG-ATC-AAC-CCA-TAC-CTG-CTA-G-3′ and (antisense) 5′-AGT-CAC-CCC-AAG-AAA-CAC-AAG-C-3′] [42], Nrf2, [(sense) 5′-AAA-CCA-GTG-GAT-CTG-CCA-AC-3′ and (antisense) 5’-GAC-CGG-GAA-TAT-CAG-GAA-CA-3′] [43], and GAPDH, [(sense) 5’-TCA-AGA-TCA-TCA-GCA-ATG-CCT-CC-3′ and (antisense) 5′-AGT-GAG-CTT-CCC-GTT-CAG-C-3′], were synthesized by MWG-Biotech AG (Ebersberg, Germany). The annealing temperature was 58 °C, 49 °C and 60 °C for PSMB5, Nrf2 and GAPDH, respectively. The PCR amplification products were analyzed and visualized by electrophoresis on 2% agarose gels, incorporating 0.01% GelRed Nucleic Acid Gel Stain (Biotium Inc. Hayward, CA, USA). The intensity of PCR products was measured using the Scion Image PC software [44] and expressed in arbitrary units (pixels). The ratio of PSMB5 or Nrf2 mRNA level to that of the house-keeping gene GAPDH was determined from the densitometric values of transcript scanning.

### Quantitative real-time PCR

Total RNA was extracted from fibroblasts using a Nucleo Spin RNA kit (Macherey-Nagel, Düren, Germany), according to the manufacturer’s instructions. The quantitative real-time PCR (qPCR) analysis was carried out at one step, using the ΚΑPA SYBR (R) FAST qPCR Master Mix (2x) kit (KAPA BIOSYSTEMS, Boston, USA), according to the manufacturer’s instructions. Assays were carried out in triplicate on a Rotor-Gene Q detection system (QIAGEN). The cycling conditions were 10 min enzyme activation at 50 °C, followed by 40 cycles at 95 °C for 5 min, 60 °C for 10 s and final 72 °C for 10 s. GAPDH was used as an internal standard. The primers used were, PSMB5: 5’-GGCAATGTCGAATCTATGAGC-3′ (sense) and 5’-GTTCCCTTCACTGTCCACGTA-3′ (antisense), and GAPDH: 5’-AGGCTGTTGTCATACTTCTCAT-3′ (sense) and 5’-GGAGTCCACTGGCGTCTT-3′ (antisense).

### Immunoblot analysis and detection of PSMB5 protein

After UVB radiation the cells were harvested, lysed in Laemmli sample buffer [45], treated with 2-mercaproethanol and then subjected to SDS-Polyacrylamide Gel Electrophoresis (SDS-PAGE) on 10% polyacrylamide gels, followed by western blotting, as previously described [46]. The membranes were incubated with rabbit polyclonal anti-PSMB5 antibody (Enzo Life Sciences) followed by secondary antibody, goat anti-rabbit IgG, conjugated with horseradish peroxidase in appropriate dilution. The immunoreactive proteins were detected by the enhanced chemiluminescence method, according to the manufacturer’s instructions (Pierce, Rockford, IL, USA). Identically, the membranes were also re-probed with a α-tubulin rabbit polyclonal antibody (Sigma-Aldrich Chemical Co., St Louis, MO). The intensity of respective protein bands was measured using the Scion Image PC software [44], expressed in arbitrary units (pixels), and the ratio PSMB5/α-tubulin was calculated.

### Statistical analysis

Data were analyzed using the unpaired Student’s t-test, with a limit of significance at *p* < 0.05. A commercial software package (Prism; GraphPad Software, San Diego, CA) was used for all data analysis and preparation of graphs.

## Results

### Expression of PSMB5 in pterygium and normal conjunctival tissues

**Fig. 1:**
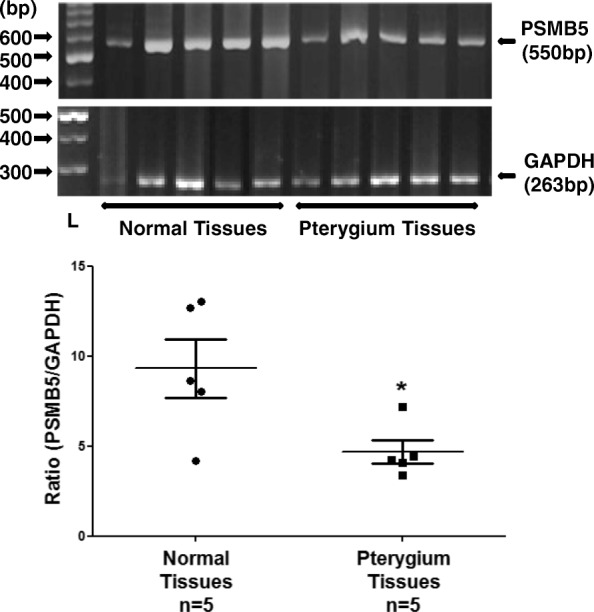
RT-PCR analysis of PSMB5 mRNA steady-state levels using total RNA from pterygium and normal conjunctiva tissues. The products for PSMB5 (550 bp) and GAPDH (263 bp) are indicated. L: DNA ladder. (*) indicates significant difference (*p* < 0.05) between normal conjunctival and pterygium tissues

The expression of PSMB5 at the mRNA level in pterygium and normal conjunctival tissues was examined using RT-PCR (Fig. 1). The expression of PSMB5 in pterygium tissues was statistically significantly lower than normal conjunctiva. This result was not attributed to differences in the age of patients with pterygium and normal conjunctiva since there were no significant differences between the age distribution and mean age of two groups of donors.

### Effects of UVB irradiation on PSMB5 expression and activity in fibroblasts

**Fig. 2:**
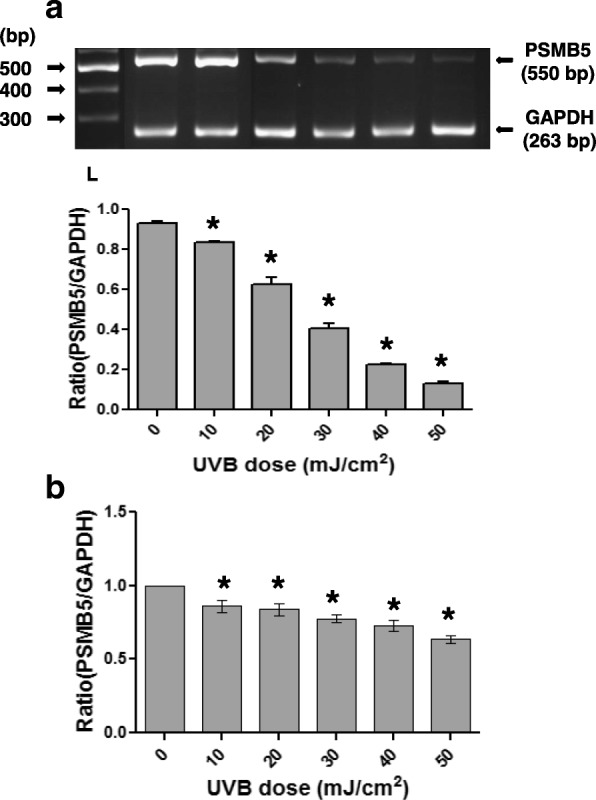
RT-PCR (**a**) and qPCR (**b**) analysis of PSMB5 mRNA steady-state levels using total RNA from pterygium and normal conjunctival fibroblasts, respectively, irradiated with different doses of UVB radiation (0–50 mJ/cm^2^). The products for PSMB5 (550 bp) and GAPDH (263 bp) are indicated. L: DNA ladder. Data represent the mean ± SD of three independent experiments. (*) indicates significant difference (p < 0.05) compared to control (non irradiated)

**Fig. 3:**
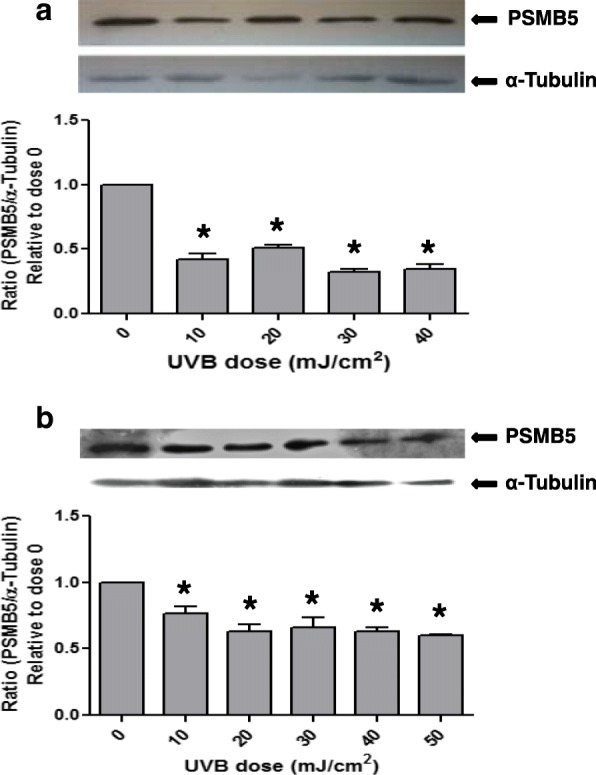
Representative western blottings of lysates of pterygium (**a**) and normal conjunctival (**b**) fibroblasts, irradiated with different doses of UVB radiation (0–50 mJ/cm^2^), using antibodies against PSMB5 or tubulin. (*) indicates significant difference (p < 0.05) compared to control (non irradiated)

Since the UVB exposure is considered as a main pathogenetic factor in pterygium disease, we examined whether the UVB irradiation has an effect on the expression of proteasome in pterygium fibroblasts, since fibroblasts produce many components of extracellular matrix and are implicated in pterygium formation. Pterygium fibroblasts were irradiated by UVB at radiation doses 0–50 mJ/cm^2^, and the expression of PSMB5 at the mRNA level was examined using RT-PCR (Fig. 2). It was observed that UVB irradiation caused a significant dose-dependent suppression of PSMB5 expression, which was higher at the doses of 40 and 50 mJ/cm^2^, 77 and 85%, respectively (Fig. 2a). The above data were further confirmed by western blotting (Fig. 3a). As shown in Fig. 3a (upper panel) only one major immunoreactive band of approximately 22 kDa, corresponding to human PSMB5, was detected in all radiation doses used. From the ratio of PSMB5 / α-tubulin, it was ascertained a radiation dose-depended decrease of PSMB5 protein production.

**Fig. 4:**
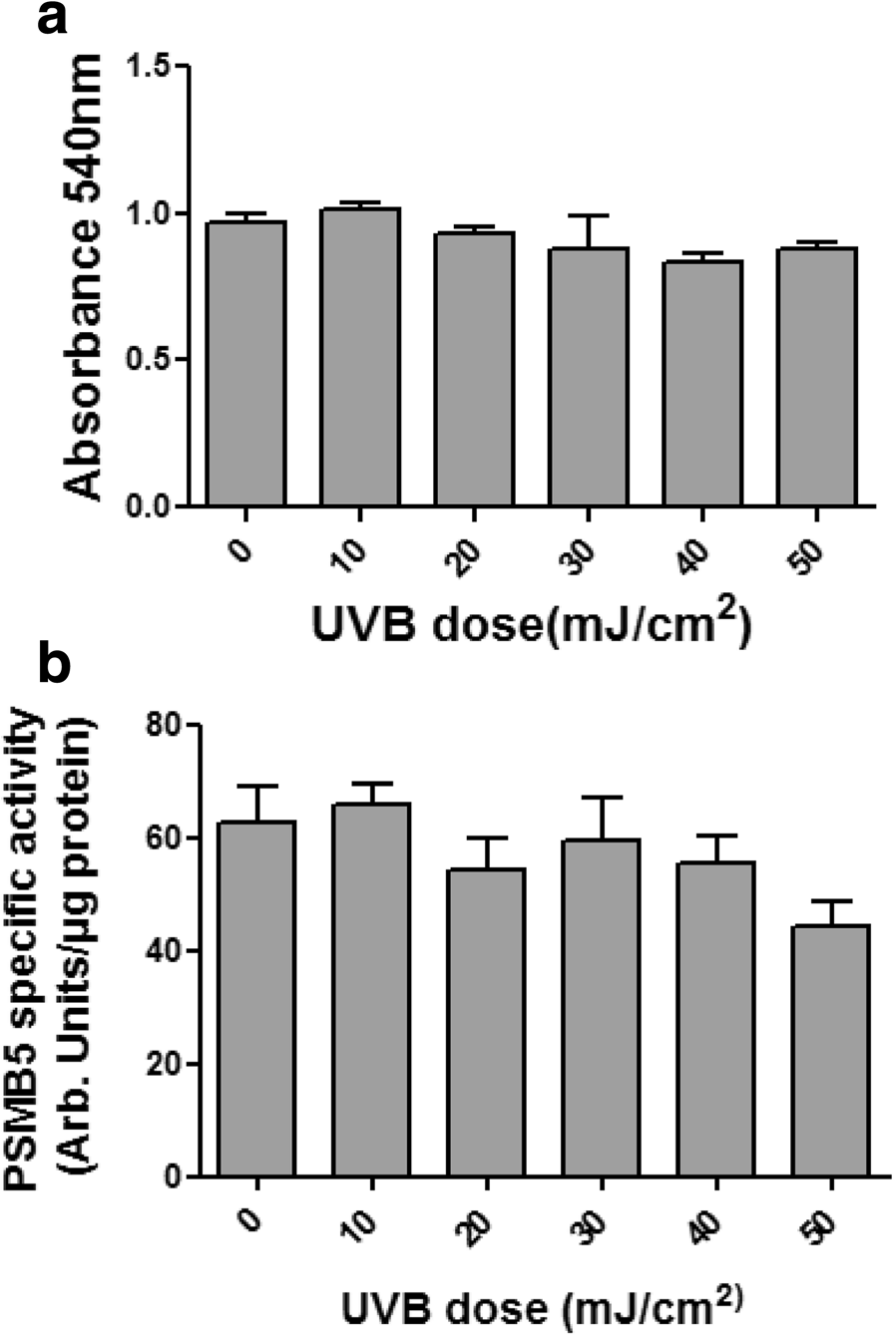
Pterygium fibroblasts viability (**a**) and proteasome chymotrypsin-like activity in these cells (**b**) after exposure to UVB radiation at different doses (0–50 mJ/cm^2^). Data represent the mean ± SD of three independent experiments. (*) indicates significant difference (p < 0.05) compared to control (non irradiated)

This effect was not the result of reduced survival of fibroblasts under UVΒ exposure, since UVΒ irradiation had very low deleterious effect on fibroblasts survival as indicated by the MTT method (Fig. 4a).

In addition UVΒ irradiation did not affect the chymotrypsin-like activity of proteasome. Upon measurement of this activity in pterygium fibroblast lysates after UVB treatment, decay of activity was not detected in any of the UVB irradiation doses used (Fig. 4b).

When normal conjunctival fibroblasts were subjected to the same treatment with UVB irradiation, a dose-dependent suppression of PSMB5 expression was also observed (Fig. 2b). However, it was lower than that caused by UVB in pterygium fibroblast (Fig. 2a), 28 and 36% at doses 40 and 50 mJ/cm^2^, respectively. This result was confirmed by western blotting (Fig. 3b). A radiation dose-depended decrease of PSMB5 protein production was also observed.

### Effects of UVB irradiation on Nrf2 expression in pterygium fibroblasts

**Fig. 5:**
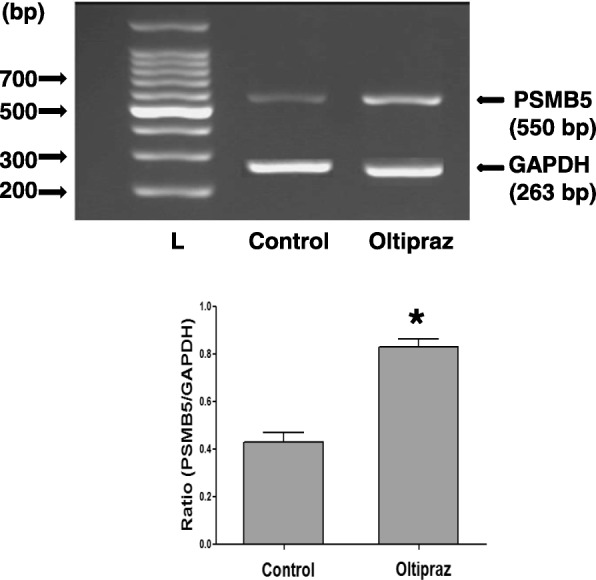
RT-PCR analysis of PSMB5 mRNA steady-state levels using total RNA from pterygium fibroblasts, cultured in the absence or presence of Oltipraz. The products for PSMB5 (550 bp) and GAPDH (263 bp) are indicated. L: DNA ladder. Data represent the mean ± SD of three independent experiments. (*) indicates significant increase (p < 0.05) compared to control

**Fig. 6:**
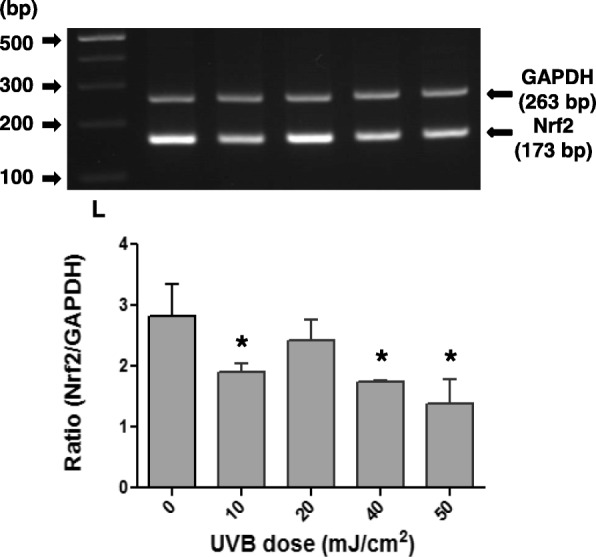
RT-PCR analysis of Nrf2 mRNA steady-state levels using total RNA from pterygium fibroblasts, irradiated with different doses of UVB radiation (0–50 mJ/cm^2^). The products for Nrf2 (173 bp) and GAPDH (263 bp) are indicated. L: DNA ladder. (*) indicates significant difference (p < 0.05) compared to control (non irradiated)

Since the expression of PSMB5 is mediated by Nrf2/ARE pathway in various cell species [27–33], we investigated whether the same occurs in pterygium fibroblasts. Pterygium fibroblasts were cultured in the presence of Oltipraz, an activator of Nrf2/ARE pathway [47, 48], and the expression of PSMB5 at the mRNA level was examined using RT-PCR (Fig. 5). The Oltipraz caused an enhancement of PSMB5 expression, indicating that the expression of this subunit in pterygium fibroblasts is indeed mediated by Nrf2/ARE pathway. Taking into account this observation, we supposed that the suppression of PSMB5 by UVB may be due to suppression of Nrf2 expression caused by UVB. It was previously reported that UVB exposure of both normal human keratinocytes and human dermal fibroblasts in vitro*,* significantly suppressed Nrf2 and Nrf2-dependent gene expression [49]. Thus, the total RNA isolated from pterygium fibroblasts, which exposed to UVB radiation at doses 0–50 mJ/cm^2^, used in RT-PCR with specific primers for Nrf2 (Fig. 6). As shown in Fig. 6, the UVB irradiation caused a dose-dependent suppression of Nrf2 expression in pterygium fibroblasts, similar but lower to the suppression achieved for PSMB5 (Fig. 2a). At doses of 40 and 50 mJ/cm^2^ the suppression of Nrf2 expression was calculated to be 35 and 50% vs 77 and 85% for PSMB5. Since the PSMB5 expression in pterygium fibroblasts is mediated by the Nrf2/ARE pathway, it would be expected that the suppression of Nrf2 expression by UVB irradiation to be about at the same levels with the suppression caused by UVB on PSMB5 expression. It seems that, although the UVB irradiation has low effect on Nrf2 expression, other factors, which may be stimulated by UVB irradiation, affect the Nrf2/ARE pathway and consequently lead to significant down-regulation of ARE-mediated PSMB5 gene expression.

### Effects of UVB irradiation on PSMB5 and Nrf2 expression in pterygium fibroblasts after pretreatment with the src kinases inhibitor PP2

**Fig. 7:**
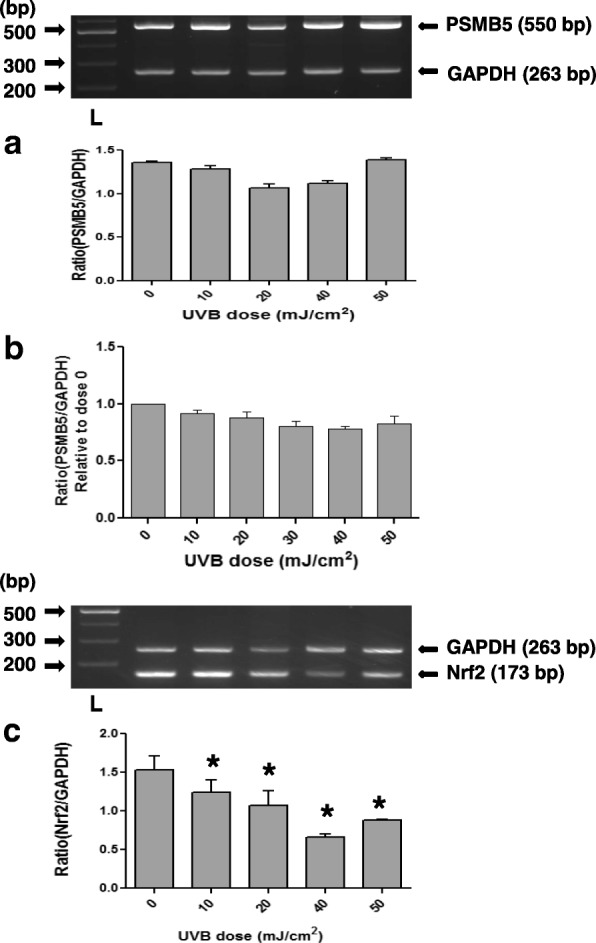
RT-PCR analysis of PSMB5 (**a**) and Nrf2 (**b**), and qPCR analysis (**c**) of mRNA steady-state levels using total RNA from pterygium and normal conjunctival fibroblasts, respectively, irradiated with different doses of UVB radiation (0–50 mJ/cm^2^), after pretreatment with PP2. The products for PSMB5 (550 bp), Nrf2 (173 bp) and GAPDH (263 bp) are indicated. L: DNA ladder. (*) indicates significant difference (*p* < 0.05) compared to control (non irradiated)

**Fig. 8:**
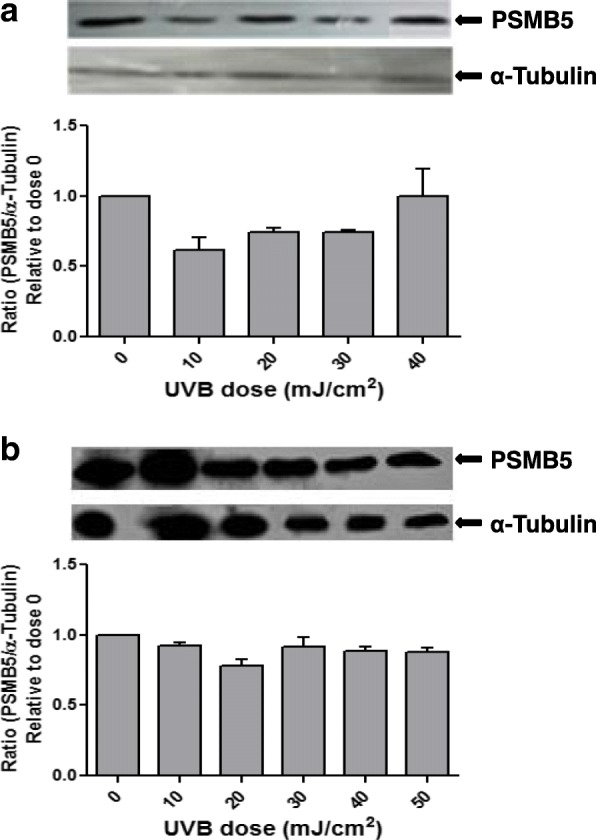
Representative western blottings of lysates of pterygium (**a**) and normal conjunctival (**b**) fibroblasts, irradiated with different doses of UVB radiation (0–50 mJ/cm^2^) after pretreatment with PP2, using antibodies against PSMB5 or α-tubulin

It has been previously reported that in mouse hepatoma cells, high dose of UVB irradiation induces the activation/nuclear localization of src family kinase Fyn, which phosphorylates the Nrf2 and leads to export out of nucleus and nuclear exclusion of this factor [50]. In order to examine whether the same applies to pterygium fibroblasts, cells were irradiated with UVB after pre-incubation with src kinase inhibitor PP2 [51], at radiation doses 0–50 mJ/cm^2^, and the expression of PSMB5 and Nrf2 at the mRNA level was examined using RT-PCR (Fig. 7). As shown in Fig. 7a, in the presence of PP2, the expression of PSMB5 remained almost at control levels (suppression only by 17.5 and 20% at doses 40 and 50 mJ/cm^2^, respectively). These results were confirmed with western blotting (Fig. 8a). From the ratio of PSMB5 / α-tubulin, it was ascertained that the production of PSMB5 protein remained almost at control levels. In contrast, following a similar procedure, it was observed that the expression of Nrf2 remained unchanged (Fig. 7c) with or without pretreatment with PP2 (Fig. 6).

The expression at the mRNA and protein levels of PSMB5 remained almost at control levels when normal conjunctival fibroblasts were irradiated after pre-incubation with PP2, as it was ascertained by qPCR and western blotting (Fig. 7b) and (Fig. 8b), respectively.

## Discussion

Although the pathogenesis of pterygium is not clearly understood, UVB radiation is considered to be the most important environmental pathogenetic factor in this disorder. Among others, it stimulates the production of reactive oxygen species (ROS) which may oxidize several components including proteins [52, 53].

The 26S proteasome is the major intracellular proteolytic system responsible for degradation of many intracellular proteins, including abnormal and oxidatively damaged proteins. Several degenerative diseases are characterized from accumulation of highly oxidized and aggregated proteins in the cytosol of cells. An abundance of evidence indicates that these aggregates block the proteasome activity, leading to cellular apoptosis and senescence [20–26]. Proteasome expression is down-regulated in senescent cells and stable over-expression of PSMB5 reverses the phenotype of senescence [54]. In this respect, the 26S proteasome is considered important for cells, protecting them from oxidative stress and senescence, and the maintenance of its expression and activity in cells may be beneficial.

From all the above observations it is expected that proteasome maybe have a key role in pterygium formation. However, the effect of UVB irradiation on proteasome expression and activity in pterygium was not studied prior to our study.

Here, we present evidence that the expression of PSMB5 in pterygium tissues is significantly lower as compared to normal conjunctival tissues, indicating that aggravating factors, such as UVB irradiation, cause down-regulation of PSMB5 expression in conjunctival cells, such us epithelial cells and fibroblasts. Taking into account the role and function of proteasome in cells, it may be suggested that the lower expression of proteasome in pterygium may be associated with its pathogenesis.

We also present evidence on the effect of UVB irradiation on PSMB5 expression and activity in pterygium fibroblasts. It was observed that UVB irradiation, which exhibited very low deleterious effect on cell viability, caused significant down-regulation of PSMB5 in pterygium fibroblasts in a radiation dose-depended mode. UVB-irradiation had the same effect, but to a lower extent, on PSMB5 expression in normal conjunctival fibroblasts, indicating that the observed effect of UVB-irradiation on pterygium fibroblasts was not the result of degeneration of these cells due to irradiation. This aspect is supported by the fact that the phenomenon was prevented in pterygium and normal fibroblasts in the presence of PP2. The differences in the extent of effect maybe due to pre-existing activation of pterygium fibroblasts from UVB-irradiation and many pro-inflammatory cytokines occurred in lesion. Different responses of pterygium and normal conjunctival fibroblasts against pro-inflammatory cytokines to produce MMPs has been previously reported [55]. Although the PSMB5 expression was suppressed, no decrease in chymotrypsin-like specific activity of this subunit was observed, indicating that proteasome is rather resistant UVB-irradiation, which is in agreement with previous results from studies in human keratinocytes and skin fibroblasts [56, 57]. In contrast, the UVA irradiation has different effects in the same cells. It causes suppression of proteasome activity but does not affect the expression of proteasome subunits, as previously reported [58, 59].

Using the Nrf2 activator Oltipraz we observed an enhancement of PSMB5 expression in pterygium fibroblasts, which is in agreement with previous studies showing that the expression of catalytic core subunit of proteasome PSMB5 bearing chemotrypsin-like activity is increased by Nrf2 through the ARE-located on the proximal promoter region of this gene [29].

Although the UVB irradiation caused suppression of Nrf2 expression, it is not the main reason for the observed high suppression of PSMB5 expression, since after pre-treatment of fibroblasts with the src kinases inhibitor PP2 and UVB exposure, the suppression in the expression of Nrf2 persisted, while the suppression in the expression of PSMB5 was prevented and its levels remained almost at the control levels, indicating that someone src kinase induced/activated by UVB irradiation is responsible for the suppression of PSMB5 expression. It has been previously reported that high dose UVB exposure of mouse hepatoma, mouse keratinocyte, and human skin fibroblast cells led to the nuclear export/exclusion of Nrf2 and decrease in ARE-mediated gene expression. In this study it was demonstrated that the kinase Fyn, a member of the src family of tyrosine kinases, mediated the nuclear exclusion of Nrf2 in response to UVB radiation [50].

It seems that the same may occur in pterygium fibroblasts exposed in UVB irradiation. However it is unknown which src kinase(s) is implicated, since the src family of tyrosine kinases consists of four members, Fyn, Lyn, Yes and Src [60]. With respect to Fyn kinase, its UVB-induced phosphorylation and nuclear localization previously reported [51]. In addition, the mechanism by which src kinase receives signals from UVB leading to the activation and nuclear export of Nrf2, remains to be clarified. However we can not exclude the possibility that the src kinase expression and activation induced by UVB may be mediated by cytokines or growth factors, the expression of which is induced by UVB. All these questions are under our consideration.

## Conclusions

In the present study we have shown that the expression of PSMB5 in pterygium is lower than that in normal conjunctiva. This may be attributed to UVB irradiation which has a suppressive effect on PSMB5 expression in pterygium fibroblasts, which in turn are known to be implicated in pterygium pathogenesis. This effect is mediated by a src kinase acting on the Nrf2/ARE pathway. As the proteasome is known to be involved in the regulation of the expression of various factors, such as metalloproteinases and their inhibitors, cytokines and collagens [58, 61–63], all of which may contribute in the formation of pterygium, it may be concluded that the implication of UVB radiation in pterygium pathogenesis is partially mediated by the suppression of proteasome expression that we also report here.

## Abbreviations

ARE: Antioxidant response element
bFGF: Basic fibroblast growth factor
DMEM: Dulbecco’s modified Eagle’s medium
DMSO: Dimethyl sulfoxide
DTT: 1,4-Dithiothreitol
FBS: Fetal bovine serum
GAPDH: Glyceraldehyde 3-phosphate dehydrogenase
IL-6: Interleukin-6
IL-8: Interleukin-8
KEAP1: Kelch-like erythroid-cell-derived protein with CNC homology (ECH)-associated protein
*MMPs*: Extracellular matrix metalloproteinases
MTT: 3-(4,5-dimethylthiazol-2-yl)-2,5-diphenyltetrazolium bromide
Nrf2: Nuclear factor (erythroid-derived 2)-like 2
Oltipraz: (5-[2-pyrazinyl]-4-methyl-1,2-dithiol-3-thione)
PDGF: Platelet-derived growth factor
PP2: 4-amino-5-(4-chlorophenyl)-7-(dimethylethyl) pyrazolo [3,4-*d*] pyrimidine
PSMB5: Proteasome subunit β5
qPCR: quantitative real-time *polymerase chain reaction*
RT-PCR: *Reverse transcription polymerase chain reaction*
SDS-PAGE: Sodium dodecyl sulfate-polyacrylamide gel electrophoresis
TGF-β: Transforming growth factor-β
TNF-α: Tumor necrosis factor-α
UVB: Ultraviolet B
